# The complete mitochondrial genome of the fall armyworm, *Spodoptera frugiperda* Smith, 1797 (Lepidoptera; Noctuidae), firstly collected in Korea

**DOI:** 10.1080/23802359.2019.1688119

**Published:** 2019-11-08

**Authors:** Bo Yoon Seo, Gwan-Seok Lee, Jonghyun Park, Hong Xi, Hyobin Lee, Jieun Lee, Jongsun Park, Wonhoon Lee

**Affiliations:** a Crop Protection Division, Department of Agro-food Safety and Crop Protection, National Institute of Agricultural Sciences, RDA, Wanju, Republic of Korea;; b InfoBoss Co., Ltd, Seoul, Republic of Korea;; c InfoBoss Research Center, Seoul, Republic of Korea;; d Department of Plant Medicine, Gyeongsang National University, Jinju, The Republic of Korea;; e Institute of Agriculture & Life Science, Gyeongsang National University, Jinju, The Republic of Korea

**Keywords:** *Spodoptera frugiperda*, mitochondrial genome, spodoptera, noctuoidae, Korea

## Abstract

The fall armyworm, *Spodoptera frugiperda*, is a serious pest in large numbers on more than 350 plant species in the world. We have determined a 15,388 bp mitogenome of *S. frugiperda* which includes 13 protein-coding genes, two ribosomal RNA genes, and 22 transfer RNAs. The base composition was AT-biased (81.3%). Phylogenetic trees present that Korean *S. frugiperda* placed in basal position of *S. frugiperda* clade. *S. frugiperda* mitochondrial genome can be used for understanding recent active migration of *S. frugiperda*.

The fall armyworm, *Spodoptera frugiperda*, is one of the most important noctuid moth pests feeds in more than 350 plant species, damaging maize, rice, sorghum, sugarcane, wheat, vegetable crops, and cotton (Montezano et al. 2018). This species is native to tropical and subtropical regions of the Americas; however, it was first reported from Africa in 2016 (Goergen et al. 2016) and from the Indian subcontinent in 2018 (Ganiger et al. 2018). In Korea, the moth was first found at corn fields of Jocheon and Gujwa, Jeju, in early June, and subsequently at many counties of Jella-do and Gyeongsang-do in June and July in this year.

Genomic DNA of *S. frugiperda* collected on *Zea mays* L. in Hwayang-myeon, Yeosu-si, Jeollanam-do, Republic of Korea in 2019 (34°68′76ʺN, 127°58′17ʺE; the specimen is stored in Gyeongsang National University, Korea accession number: Coll#HB101) was extracted using DNeasy Brood &Tissue Kit (QIAGEN, Hilden, Germany). HiSeqX was used for sequencing (Macrogen Inc., Korea). Filtering, *de novo* assembly, and gap-filing processes were done by Velvet 1.2.10 (Zerbino and Birney 2008), Trimmomatic 0.33 (Bolger et al. 2014), SOAPGapCloser 1.12 (Zhao et al. 2011), BWA 0.7.17 (Li 2013), SAMtools 1.9 (Li et al. 2009). Geneious R11 11.1.5 (Biomatters Ltd, Auckland, New Zealand) and ARWEN (Laslett and Canbäck 2008) were used to annotate *S. frugiperda* based on alignment of other *Spodoptera* mitochondrial genomes.

*S. frugiperda* mitochondrial genome (Genbank accession is MN385596) is 15,388 bp long, the second longest among four *S. frugiperda* mitochondrial genomes. Its GC ratio is 18.7%, the lowest among four. It contains 13 protein-coding genes (PCGs), two rRNAs, and 22 tRNAs. Gene order of *S. frugiperda* is identical to other *Spodoptera* mitochondrial genomes, presenting conserved gene order of Noctuoidea mitochondrial genomes (Liu et al. 2016).

Based on alignments against three *S. frugiperda* mitochondrial genomes (NC_027836, KU877172, and KJ778892), 43 insertions or deletions (INDELs) and 275 single nucleotide polymorphisms (SNPs), 49 INDELs and 351 SNPs, and 74 INDELs and 247 SNPs were identified, respectively. Amounts of these intraspecies sequence variations are similar to that of *Chilo suppressalis* (79 SNPs and 291 INDELs) (Park, Xi, et al. 2019) and more than those of *Laodelphax striatellus* (140 and 40 SNPs and 166 and 118 INDELs) (Park, Jung, et al. 2019), *Nilaparvata lugens* (112 SNPs and 59 INDELs) (Park, Kwon, et al. 2019) and *Aphis gossypii* (61 SNPs and 3 INDELs; Park, Xi et al., 2019).

We inferred the phylogenetic relationship of 31 Noctuidae mitochondrial genomes including *Spodoptera* species and *Lymantria dispar* as an outgroup. Multiple sequence alignment was conducted by MAFFT 7.388 (Katoh and Standley 2013) using concatenated alignments of 13 PCGs. Bootstrapped maximum likelihood and neighbor joining trees were constructed using MEGA X (Kumar et al. 2018). Korean *S. frugiperda* is clustered with other three *S. frugiperda* (Figure 1), and all mitogenomes of *Spodoptera* genus form one clade, presenting monophyletic manner (Figure 1). Four mitogenomes of *S. frugiperda* showed intraspecies variations, suggesting the new mitogenome analyzed in this study will be helpful to understand geographical genetic variations of *S. frugiperda*.

**Figure 1.**
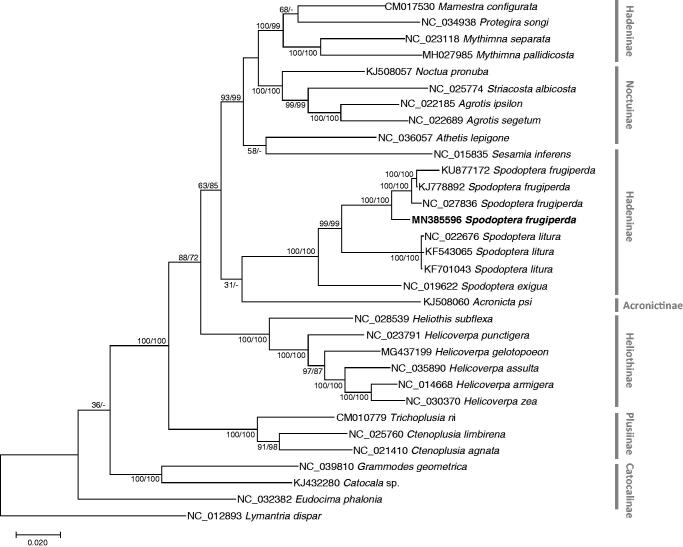
Maximum likelihood (bootstrap repeat is 1000) and neighbor-joining (bootstrap repeat is 10,000) phylogenetic tree of all *Spodoptera* mitochondrial genomes: *Spodoptera frugiperda* (MN385596: this study, NC_027836, KJ778892, and KU877172), *Spodoptera exigua* (NC_019622), *Spodoptera litura* (NC_022676, KF701043, and KU877172), 23 Noctuidae species: *Acronicta psi* (KJ508060), *Agrotis ipsilon* (NC_022185), *Agrotis segetum* (NC_022689), *Athetis lepigone* (NC_036057), *Catocala* sp. (KJ432280), *Ctenoplusia agnate* (NC_021410), *Ctenoplusia limbirena* (NC_025760), *Eudocima phalonia* (NC_032382), *Grammodes geometrica* (NC_039810), *Helicoverpa armigera* (NC_014668), *Helicoverpa assulta* (NC_035890), *Helicoverpa gelotopoeon* MG437199), *Helicoverpa punctigera* (NC_023791), *Helicoverpa zea* (NC_030370), *Heliothis subflexa* (NC_028539), *Mamestra configurata* (CM017530), *Mythimna pallidicosta* (MH027985), *Mythimna separata* (NC_023118), *Noctua pronuba* (KJ508057), *Protegira songi* (NC_034938), *Sesamia inferens* (NC_015835), *Striacosta albicosta* (NC_025774), *Trichoplusia ni* (CM010779), and *Lymantria dispar* (NC_012893) as an outgroup. Phylogenetic tree was drawn based on maximum likelihood phylogenetic tree. The numbers above branches indicate bootstrap support values of maximum likelihood and neighbor joining phylogenetic trees, respectively.
